# Robotic versus Laparoscopic Emergency and Acute Care Surgery: Redefining Novelty (RLEARN): feasibility and benefit of robotic cholecystectomy for acute cholecystitis at a level 1 trauma center

**DOI:** 10.1136/tsaco-2024-001522

**Published:** 2024-12-27

**Authors:** Joshua Klein, Mekedes Lemma, Kartik Prabhakaran, Aryan Rafieezadeh, Jordan Michael Kirsch, Gabriel Rodriguez, Ilyse Blazar, Anna Jose, Bardiya Zangbar

**Affiliations:** 1 Surgery, Westchester Medical Center, Valhalla, New York, USA

**Keywords:** cholecystectomy, laparoscopic, acute care surgery

## Abstract

**Background:**

This study aims to compare outcomes of robotic cholecystectomy (RC) versus laparoscopic cholecystectomy (LC) in the setting of a level 1 trauma center.

**Methods:**

We performed a retrospective study of our hospital data (2021–2024) on patients who underwent LC or RC. Using a previously validated intraoperative grading system, four grades of cholecystitis were defined as mild (A), moderate (B), severe (C), and extreme (D). Outcomes were operative times and rates of conversion to open surgery.

**Results:**

In total, 260 patients (n=130 RC and n=130 LC) were included. Patients were primarily female (69.2%), with mean age of 47±18.3 years. The majority of cases had grade B cholecystitis (41.2%). Patients undergoing RC had lower operative times compared with LC in grade B (101.87±17.54 vs 114.96±29.44 min, p=0.003) and grade C (134.68±26.97 vs 152.06±31.3 min, p=0.038). Conversion rate to open cholecystectomy were similar in both groups (p=0.19).

**Conclusion:**

RC had similar results as LC in terms of operative time and in fact has significantly lower operative time in patients with grade B and grade C cholecystitis.

**Level of evidence:**

Level III—retrospective study.

**WHAT IS ALREADY KNOWN ON THIS TOPIC**

- Robotic cholecystectomy (RC) is a relatively new technique compared with laparoscopic cholecystectomy (LC); however, there is much to discover regarding differences of the two methods in terms of operative characteristics.

**WHAT THIS STUDY ADDS**

- This study aims to determine if the use of robotic-assisted surgery results in decreased operative time and improved clinical outcomes across the different grades of cholecystitis compared with LC.

**HOW THIS STUDY MIGHT AFFECT RESEARCH, PRACTICE OR POLICY**

- The results of our study influence the patient’s outcomes that could, in turn, be used in improvement of patient’s healthcare.

## Introduction

 Since the first laparoscopic cholecystectomy (LC) performed in 1985, LC has become the standard of care for patients requiring cholecystectomy.^1 2^ With the increasing applicability of robotic-assisted surgery throughout surgical specialties, general and acute care surgeons have begun using this technology to perform a variety of operations. Whereas robotic surgery has largely gained popularity for elective surgical procedures, there has been more recently a push to perform emergency general surgery operations robotically as well.

Robotic cholecystectomy (RC) in the setting of a busy level 1 trauma and tertiary referral hospital has been scrutinized for potentially prolonged operative times.^3^ Other criticisms include the questionable added benefit to the patient, considering the existing minimally invasive nature of LC, as well as the learning curve faced by surgeons adopting robotic procedures.^4^ Advocates of RC, however, note that the robotic surgical system allows for enhanced three-dimensional visualization, increased instrument range of motion, and improved surgical precision. Fluorescence cholangiography can be performed during RC without adding to the operative time, whereas conventional cholangiography can potentially prolong the operation.^5^

While previous studies have investigated operative time, conversion to open surgery, mortality, and cost, there have been no studies comparing outcomes between RC and LC based on degree of cholecystitis. This study aims to determine if the use of robotic-assisted surgery results in decreased operative time and improved clinical outcomes across the different grades of cholecystitis compared with LC.

## Methods

We performed a 3-year (from January 2021 to March 2024) retrospective study of our hospital database that is an American College of Surgeons-verified level 1 trauma and tertiary referral medical center. We included patients 18 years or older who underwent LC or RC.

All surgeries were performed by six surgeons who were proficient in performing LC and RC (surgeons were anonymized and were labeled by letters A–F). Patients were offered LC versus RC based on the availability of the Da Vinci Robotic system, availability of robotically trained surgeon (as there were more than six surgeons in our institute), and patient preference. Patients younger than 18 years of age and cases in which a cholecystectomy was performed in combination with another operation were excluded. Patients who underwent partial cholecystectomy or conventional cholangiography were also excluded. At our institution, the decision to use fluorescence cholangiograms is made at the surgeon’s discretion and is not a standard practice for all cholecystectomies. Fluorescence cholangiography can be used during both RC and LC without significantly impacting operative time. In contrast, conventional cholangiography is an additional procedure and can prolong the procedures and was a reason for excluding those cases from our analysis to ensure consistency and focus on the primary outcomes.

The most immediate historical matching number of patients undergoing LC during, and prior to implementation of the RC program, were selected as the control group.

Since this study was conducted in a teaching hospital, it is important to note that attending surgeons were consistently present and scrubbed throughout the entirety of the surgical procedures. Additionally, surgical trainees participated commensurate to their level of experience, regardless of surgical approach. All surgeons had significant experience in laparoscopic surgery, with each having over 3 years of operative experience as attending surgeons. A minimum of 25 robotic cases were required for proficiency. However, we should note that by the time data collection for this study commenced in 2024, all six surgeons had already been certified and had extensive experience with this surgery. Specifically, each surgeon had performed >30 RC cases before the study began.

Notably, cases were not postponed pending the availability of the robotic system.

For evaluating the severity of cholecystitis, we used *World Journal of Emergency Surgery* (WJES) (table 1) and Parkland grading scale for acute cholecystitis (PGS).

**Table 1:** Operative grading system for cholecystitis severity (WJES)

Item	Score
GB appearance	
Adhesion <50% of GB	1
Adhesion burying GB	3
	Max 3
Distention/Contraction	
Distended GB (or contracted shriveled GB)	1
Unable to grasp with atraumatic laparoscopic forceps	1
Stones ≥1 cm impacted in Hartman’s pouch	1
Access	
BMI >30	1
Adhesion from previous surgery limiting access	1
Severe sepsis/complications	
Bile or pus outside GB	1
Time to identify cystic artery and duct >90 min	1
	Total max 10
Degree of difficulty	
A—mild	<2
B—moderate	2–4
C—severe	5–7
D—extreme	8–10

BMIbody mass indexGB, gallbladderWJES*World Journal of Emergency Surgery*

It should be noted that surgeons were aware of the possibility of conducting this study and committed to produce very detailed operative dictations which included components of WJES score during each surgery by the operating surgeon and was documented in the operative notes for each patient. However, the vocabulary and template for operative notes were not uniform. Also, two independent physician reviewers graded the degree of cholecystitis using the WJES scoring system based on the text within the operative dictations and other data within the patient’s medical record and any disparity between was adjudicated by a third physician reviewer. Adjudication was required in 18 cases, comprising 12 cases in the robotic group and six cases in the laparoscopic group. For adjudication, the grade of cholecystitis that was in the majority among the three physician reviewers was used as the final grade. There were no instances where the grade of cholecystitis determined by all three were different.

PGS is a classification system used to assess the severity of acute cholecystitis based on intraoperative findings.^6 7^

- Grade 1 PGS is defined as completely normal appearing gallbladder and no adhesions.
- Grade 2 PGS is defined as adhesions restricted to the neck or lower of the gallbladder, otherwise normal gallbladder.
- Grade 3 PGS is defined as presence of ANY of: hyperemia, pericholecystic fluid, adhesions to the body, distended gallbladder.
- Grade 4 PGS is defined as presence of ANY of: adhesions obscuring majority of gallbladder, grade I–III with abnormal liver anatomy, intrahepatic gallbladder, or impacted stone (Mirrizi).
- Grade 5 PGS is defined as presence of ANY of: perforation, necrosis, inability to visualize the gallbladder due to adhesions.

PGS scores were retrospectively collected through chart review.

Operative times (incision to close), sex, and age were extracted. Our primary outcome variable was operative time (incision to close). The secondary outcome was the conversion rate to open surgery. We should note that none of the surgeons have transitioned their practice to robotic surgery completely, and all acute care surgeons continued to perform laparoscopic cholecystectomies. We also assessed occurrence of other complications including bile leak, common bile duct injuries, surgical site infection, readmission and thromboembolic events (such as deep vein thrombosis or pulmonary embolism) as well as mortality. Apart from conversion to open surgery, there were no instances of mortality or other complications in this study. Patient’s follow-up postoperative visits within 10–14 days were also reviewed and complications were recorded.

We used SPSS V.24 (IBM, Armonk, New York, USA) for data analysis. To compare the differences in mean operative time between the two procedures, we conducted an independent samples t-test. Sex, WJES grade, and PGS between the two procedures were compared using χ^2^ tests. We compared the mean operative times within WJES and PGS grades using independent samples t-tests to evaluate if operative times differed by procedure. Additionally, we examined how times differed between grades within a procedure using independent samples t-tests. We considered a p value <0.05 as the threshold for statistical significance.

## Results

In total, 260 patients were included in this study, divided into two groups of LC and RC, each having 130 patients. There were no significant differences in number of cases done by each surgeon (A–F) (p=0.219). It was also shown that there were no significant differences in the number of patients in each severity grade, as divided by the surgeons (p>0.05, using both WJES and PGS) (tables2  3). We observed no significant difference between the two groups regarding operative time, as divided by the surgeons (p>0.05) (table 4).

**Table 2:** Severity of cases based on WJES grade divided by each surgeon

Surgeon	Group								P value*
	Laparoscopic (n=130)				Robotic (n=130)				
	WJES grade (n (%))				WJES grade (n (%))				
	A	B	C	D	A	B	C	D	
A	13 (48.1)	9 (33.3)	5 (18.5)	0	8 (40)	8 (40)	4 (20)	0	0.85
B	6 (46.2)	5 (38.5)	0	2 (15.4)	10 (37)	8 (26.9)	7 (25.9)	2 (7.4)	0.229
C	7 (50)	3 (21.4)	3 (21.4)	1 (7.1)	4 (30.8)	6 (46.2)	2 (15.4)	1 (7.7)	0.576
D	4 (40)	5 (50)	1 (10)	0	4 (66.7)	2 (33.3)	0	0	0.504
E	19 (38)	24 (48)	6 (12)	1 (2)	14 (28.6)	22 (44.9)	7 (14.3)	6 (12.2)	0.214
F	5 (31.3)	9 (56.3)	1 (6.3)	1 (6.3)	4 (26.7)	6 (40)	2 (13.3)	3 (20)	0.569

There were no significant differences between the surgeons regarding number of cases (pp=0.219).

* Comparing number of cases based on grades for each surgeon, using chi-squareχ2 test.

WJES, *World Journal of Emergency Surgery*

**Table 3:** Severity of cases based on PGS grade divided by each surgeon

Surgeon	Group										P value*
	Laparoscopic (n=130)					Robotic (n=130)					
	PGS (n (%))					PGS (n (%))					
	1	2	3	4	5	1	2	3	4	5	
A	13 (48.1)	9 (33.3)	2 (7.4)	3 (11.1)	0	8 (40)	8 (40)	3 (15)	1 (5)	0	0.696
B	6 (46.2)	5 (38.5)	0	0	2 (15.4)	10 (37)	8 (26.9)	4 (14.8)	3 (11.1)	2 (7.4)	0.364
C	7 (50)	3 (21.4)	2 (14.3)	1 (7.1)	1 (7.1)	4 (30.8)	6 (46.2)	1 (7.7)	1 (7.7)	1 (7.7)	0.714
D	4 (40)	5 (50)	0	1 (10)	0	4 (66.7)	2 (33.3)	0	0	0	0.504
E	19 (38)	24 (48)	3 (6)	3 (6)	1 (2)	14 (28.6)	22 (44.9)	5 (10.2)	2 (4.1)	6 (12.2)	0.277
F	5 (31.3)	9 (56.3)	0	1 (6.3)	1 (6.3)	4 (26.7)	6 (40)	2 (13.3)	0	3 (20)	0.321

There were no significant differences between the surgeons regarding number of cases (pp=0.219).

* Comparing number of cases based on grades for each surgeon, using χ2 test.

PGS, Parkland grading scale for acute cholecystitis

**Table 4:** Number of cases and operation times divided by each surgeon

Surgeon	Group				P value*
	Laparoscopic (n=130)		Robotic (n=130)		
	Number of cases (%)	Operation time (min) (mean±SD)	Number of cases	Operation time (min) (mean±SD)	
A	27 (20.8)	107.19±33.57	20 (15.4)	100.55±35.44	0.516
B	13 (10)	108.15±38.46	27 (20.8)	110.15±30.12	0.429
C	14 (10.8)	105.79±41.83	13 (10)	108.31±50.86	0.889
D	10 (7.7)	119.1±44.28	6 (4.6)	106.17±27.31	0.532
E	50 (38.5)	102.84±38.98	49 (37.7)	105.14±33.56	0.754
F	16 (12.3)	121.31±35.103	15 (11.5)	105±36.43	0.214

There were no significant differences between the surgeons regarding number of cases (pp=0.219).

* Comparing operation time for each surgeon using independent t-test.

There was no statistically significant difference in age, sex, WJES grade, PGS, and mean operative time when comparing the LC versus RC (table 5). Females comprised the majority of the patient population, with males making up 29.2% and 32.3% of the patients in the LC and RC groups, respectively. There were no significant differences between the two groups regarding duration of operation (p=0.612). Patients who received LC had a mean operative time of 108.12±37.96 min, while those who received RC had a mean time of 105.82±34.78 min.

**Table 5:** Descriptive analysis of patient demographic data

Characteristic	Overall (n=260)	Laparoscopic (n=130)	Robotic (n=130)	P value
Age (mean±SD)	47±18.3	45.33±19.48	48.66±17.08	0.144*
Age group (n (%))				
18–34 years	74 (28.8%)	45 (34.9%)	29 (22.7%)	0.06†
35–44 years	47 (18.3%)	26 (20.2%)	21 (16.4%)	
45–64 years	94 (35.6%)	35 (26.3%)	59 (44.5%)	
65+ years	45 (17.3%)	24 (18.6%)	21 (16.4%)	
Sex (n (%))				
Female	180 (69.2%)	92 (70.8%)	88 (67.7%)	0.591†
Male	80 (30.8%)	38 (29.2%)	42 (32.3%)	
WJES grade (n (%))				
A	98 (37.7%)	54 (41.5%)	44 (33.8%)	0.177†
B	107 (41.2%)	55 (42.3%)	42 (40%)	
C	38 (14.6%)	16 (12.3%)	22 (16.9%)	
D	17 (6.5%)	5 (3.8%)	12 (9.2%)	
PGS (n (%))				
1	98 (37.7%)	54 (41.5%)	44 (33.8%)	0.128†
2	107 (41.2%)	55 (42.3%)	42 (40%)	
3	22 (8.5%)	7 (5.4%)	15 (11.5%)	
4	16 (6.2%)	9 (6.9%)	7 (5.4%)	
5	17 (6.5%)	5 (3.8%)	12 (9.2%)	
Total operative time (min) (mean±SD)	106.97±36.35	108.12±37.96	105.82±34.78	0.612*

* Two -sample Tt-test.

† Pearson’s Chi-squareχ2 test.

PGS, Parkland grading scale for acute cholecystitisWJES, *World Journal of Emergency Surgery*

**Figure 1:**
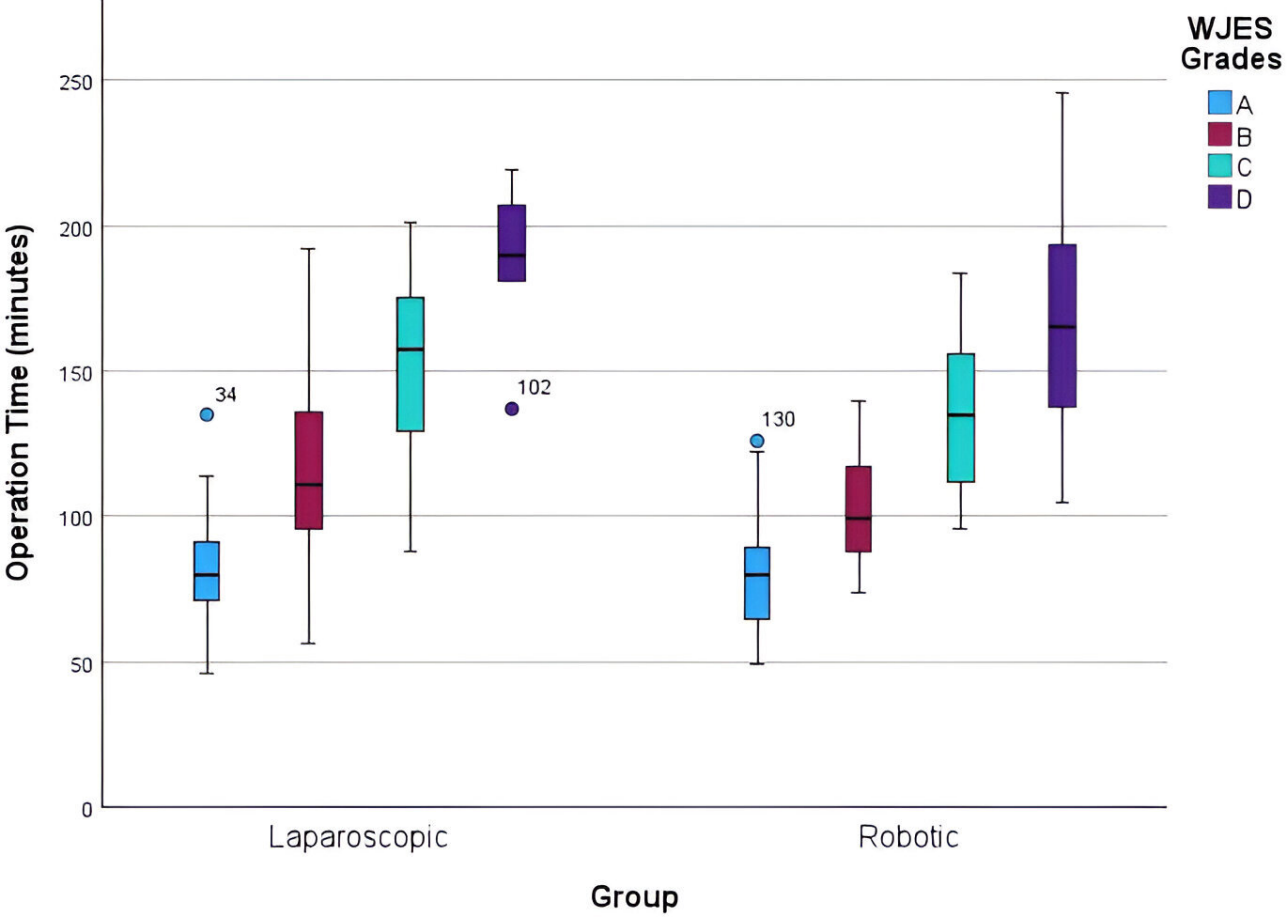
Box plot of operative times by *World Journal of Emergency Surgery* (WJES) grade.

When considering the WJES grade in comparing LC with RC, we found that patients with a grade B or C cholecystitis showed differences in mean operative times (p=0.003 and p=0.038, respectively), whereas patients with a grade A or D cholecystitis did not have statistically significant difference in mean operative times (p=0.73 and p=0.33, respectively) (table 6). Patients with a WJES grade of B or C cholecystitis who received LC had a mean operative time that was 13.09 min and 17.38 min longer than RC, respectively. We also observed an upward trend in operative times in both LC and RC when accounting for WJES and PGS grades (figures1  2). There was no statistically significant difference in the conversion to open surgery between the LC and RC groups (p=0.19) (table 6).

**Table 6:** Outcomes of cholecystectomy patients by surgical modality and WJES grade

Outcomes	WJES grade	Laparoscopic cholecystectomy	Robotic cholecystectomy	P value
Total operative timeMean±SD (number of cases)	A	80.83±17.25 (n=54)	79.66±17.33 (n=44)	0.73*
	B	114.96±29.44 (n=55)	101.87±17.54 (n=52)	0.003*
	C	152.06±31.3 (n=16)	134.68±26.97 (n=22)	0.038*
	D	186.8±31.49 (5)	166±42.06 (n=12)	0.33*
Conversion rate	A	0 (n=54)	0 (n=44)	–
	B	0 (n=55)	0 (n=52)	–
	C	0 (n=16)	0 (n=22)	–
	D	2 (n=5)	1 (n=12)	0.19†

* Two -sample Tt-test.

† Fischer’s exact test.

WJES, *World Journal of Emergency Surgery*

**Figure 2:**
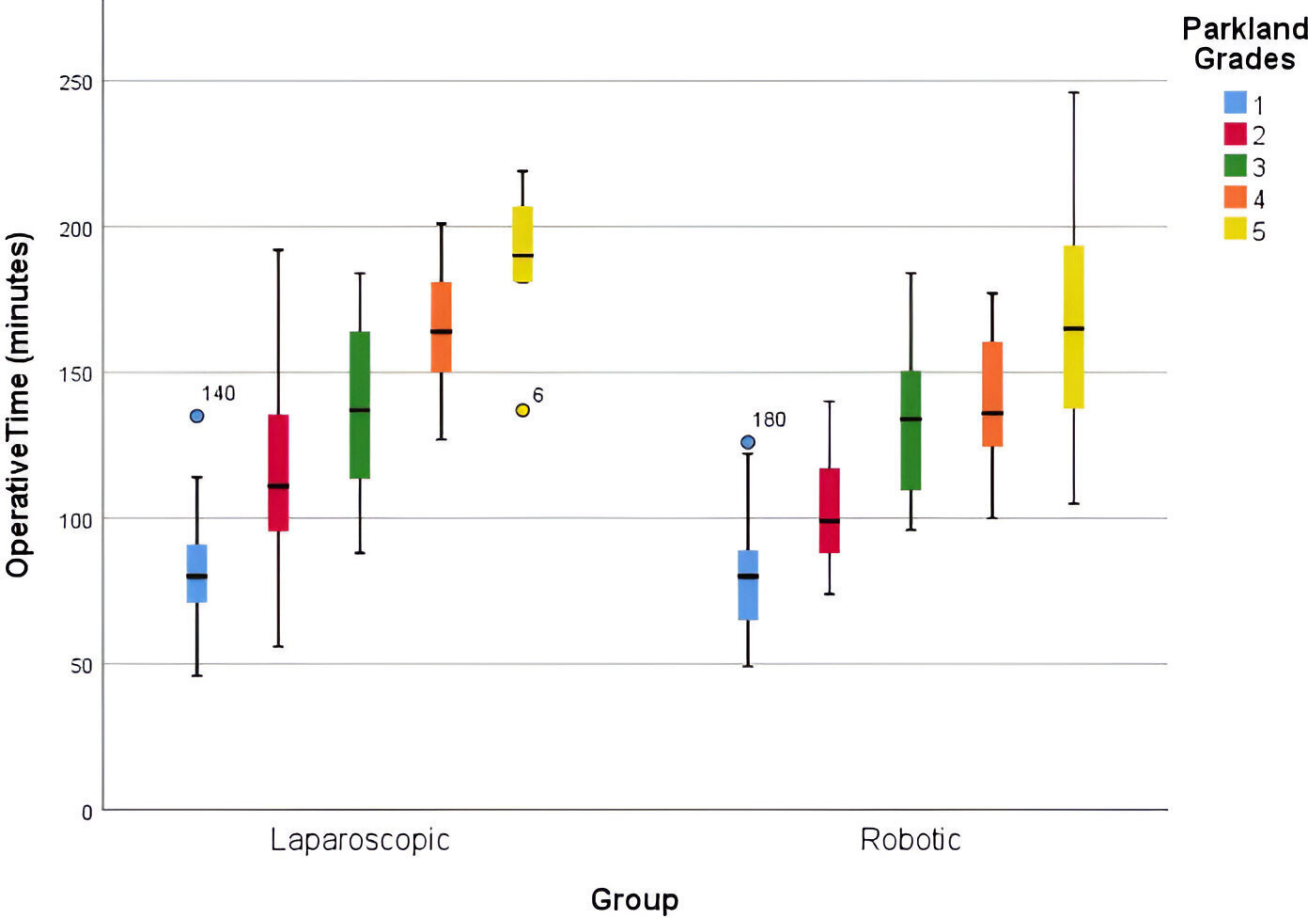
Box plot of operative times by Parkland grading scale.

In this study, there were three conversions to open cholecystectomy: one case occurred during RC (0.8%) and two cases during LC (1.5%). All patients had grade D or 5 cholecystitis. The reason for conversion to open surgery in the RC case was uncontrollable bleeding from the liver bed that could not be managed with robotic instruments. For the two LC cases, the conversions to open surgery were necessitated by the inability to delineate pertinent anatomical structures. Despite the use of fluorescence cholangiography in both patients prior to conversion, the anatomical landmarks could not be adequately visualized.

We also used PGS to compare the outcomes between the two groups. We observed that the mean operative time was significantly higher in LC in grade 2 (p=0.003), while there were no other significant differences between the two groups in other grades of severities. There was also no statistically significant difference in conversion rates between the two groups (table 7).

**Table 7:** Outcomes of cholecystectomy patients by surgical modality and WJES grade

Outcomes	PGS grade	Laparoscopic cholecystectomy	Robotic cholecystectomy	P value
Total operative timeMean±SD (number of cases)	1	80.83±17.25 (n=54)	79.66±17.33 (n=44)	0.73*
	2	114.96±29.44 (n=55)	101.87±17.54 (n=52)	0.003*
	3	137.71±34.68 (n=7)	132±27.24 (n=15)	0.678*
	4	163.22±24.79 (n=9)	140.43±27.53 (n=7)	0.104*
	5	186.8±31.49 (5)	166±42.06 (n=12)	0.33*
Conversion rate	1	0 (n=54)	0 (n=44)	–
	2	0 (n=55)	0 (n=52)	–
	3	0 (n=7)	0 (n=15)	–
	4	0 (n=9)	0 (n=7)	–
	5	2 (n=5)	1 (n=12)	0.19†

* Two -sample Tt-test.

† Fischer’s exact test.

PGS, Parkland grading scale for acute cholecystitisWJES*World Journal of Emergency Surgery*

There were no complications, including bile leak, common bile duct injuries, surgical site infections, readmissions, thromboembolic events, or mortality in our study population after chart review, including postoperative short-term follow-up visits.

## Discussion

This study found that in patients with WJES grade B and grade C cholecystitis, operative times were shorter when surgery was performed robotically as opposed to laparoscopically. By using PGS, the operative time was lower in RC compared with LC in grade 2. Whereas previous studies have compared operative times between LC and RC, no prior study has graded the degree of cholecystitis encountered intraoperatively and compared LC with RC.^38^,^10^ In comparison with traditionally studied presurgical variables such as those used in the Tokyo guidelines, intraoperative grading of cholecystitis offers the benefit of being able to compare patients with similar operative complexity and determine if robotic-assisted surgery is beneficial in a certain subset of patients.^11^

The intraoperative scoring system developed by Sugrue *et al* aimed to standardize the definitions and degree of cholecystitis at the time of operation.^12^ Analysis of our LC and RC operative times show an increasing median operative time regardless of operative modality performed as the grade of cholecystitis increases lending support for the proposed operative classification system.

An operation on patients with grade A cholecystitis should theoretically have a lower complexity level than patients with higher grades of cholecystitis. As surgery for grade A cholecystitis will generally be more straightforward, the enhanced range of motion and improved surgical precision offered by the robotic platform may not be needed to be used maximally, which is why we do not see a significant decrease in operative time between RC and LC in these operations. Similar pattern was observed when using PGS as the grading system in this study.

On the other end of the spectrum, grade D cholecystitis, similar to grade 5 based on PGS, should have the highest operative complexity. While the three-dimensional visualization, instrument precision, and increased instrument range of motion may be beneficial to the surgeon, grade D or grade 5 cholecystitis operations frequently encounter a large number of adhesions, difficult to grasp tissue, and gangrenous gallbladders that would be difficult regardless of the modality used to perform the cholecystectomy. While the mean and median operative times of grade D and grade 5 cholecystitis were overall lower in the RC group, the times did not reach statistical significance likely secondary to the smaller number of patients categorized as having this grade of cholecystitis as opposed to lesser grades.

Prior literature investigating conversion from LC to open cholecystectomy have reported a wide range of conversion rates, between 2.4% and 19%.^13^,^15^ In a recent study by Lunardi *et al*, the rate of conversion to open surgery was 1.7% in RC and 3% in LC.^16^ In our study, the conversion rate was 4% in the LC group and 1.3% in the RC group, which is consistent with the previous studies. All conversions to open cholecystectomy were performed in patients who had grade D or grade 5 cholecystitis, with the most frequently cited reason for conversion within operative reports being inability to delineate pertinent anatomical structures.

Data regarding physical examination findings, serum blood testing, and imaging characteristics were not analyzed, as these cannot fully predict the severity of cholecystitis and operative difficulty. They are believed to be less impactful for this study than the grade of cholecystitis as measured by the WJES scoring system.^17^ Current literature has yet to uniformly identify and validate physical examination findings, laboratory values, and preoperative ultrasonographic characteristics that can accurately predict the difficult cholecystectomy. In a study by Di Buono *et al*, radiographic findings of gallbladder wall thickening, absence/wall irregularity, and hydropic gallbladder were predictive factors of difficult cholecystectomy.^18^ However, in a prospective, non-randomized trial by Stanisic *et al*, the authors found that only stone impaction identified on ultrasound had predictive values for determining difficult cholecystectomy.^19^ Given the variability in the literature regarding identification of preoperative characteristics that predict a difficult cholecystectomy, we believe that intraoperative findings are more applicable in determining the true degree of cholecystitis and the difficulty of cholecystectomy.

### Limitations

Our study is limited due to the retrospective and non-randomized study design, and non-uniform operative dictation template. Due to the relatively small number of cases and the overall scarcity of bile duct injuries and bile leak, the study lacks sufficient power to adequately assess these particular complications. However, bile duct injury did not occur in either of our patient population groups. The same is true for surgical site infection. Other studies have reported higher rates of common bile duct injuries in RC; however, the results of those studies have been controversial.^20^ Another point is that patients undergoing partial cholecystectomy were excluded from our study due to the lack of detailed reporting on the extent of gallbladder resection in the operation notes. This exclusion was necessary to ensure the reliability and validity of our outcomes, as the variable nature of partial cholecystectomy could have introduced significant confounding factors that would obscure the comparison between the two techniques.

Another limitation is related to surgical resident involvement in the case, as is true for most teaching institutions. The resident scrubbed in for the surgery was not a constant and depended on surgical case assignment. The resident’s skill level, familiarity with the robotic surgical system, and the amount of autonomy given may have affected operative times.

Although selection bias is possible due to retrospective nature of the study, we believe the implemented intraoperative scoring system mitigates selection bias as seen by similar grades of cholecystitis. Due to the retrospective nature of the study, randomization was not feasible. Another limitation is the potential selection bias introduced by the varying levels of training among operating room staff. The availability of trained robotic staff, especially during night shifts, or availability of the robots based on the service that had previously booked them, influenced the choice between RC and LC, potentially affecting the study outcomes. However, the most appropriate comparison group available was established by comparing robotic cases with the last laparoscopic cases performed by the same surgeons prior to the introduction of robotic surgery. An anticipated bias was that the surgeons’ most recent laparoscopic cases would represent their most experienced operations in laparoscopy, while their initial robotic cases would reflect a learning curve. Nonetheless, the results indicate that despite this potential bias, robotic surgery had similar results and, in some instances, even exhibited superiority over laparoscopic surgery in terms of operating time.

As this study was performed at a level 1 tertiary referral hospital, we receive a higher number of patients transferred to our institution for surgical procedures than smaller surrounding community and critical access hospitals. While the reason for transfers are wide-ranging, this study may have a higher proportion of patients with higher grades of cholecystitis than would be seen in community hospitals or elective practice.

## Conclusion

RC can be the preferred surgical approach a busy level 1 trauma and tertiary referral/teaching hospital. RC has lower operative times in relatively complicated cases with a similar rate of conversion to open surgery when compared with LC. Prospective studies with larger number of cases are warranted to compare rare complications.

## Data Availability

Data are available on reasonable request.
